# The in vitro elution characteristics of vancomycin from the ligament augmentation and reconstruction system

**DOI:** 10.1002/jeo2.70104

**Published:** 2024-12-03

**Authors:** Yong Luo, MingYang Zou, XinTao Zhang, SuFen Ye, XianCheng Huang, JiaTong Li, Tian You

**Affiliations:** ^1^ Sports Medicine and Rehabilitation Center Peking University Shenzhen Hospital Shenzhen China; ^2^ Southern University of Science and Technology Shenzhen China; ^3^ Weifang Medical University Weifang China; ^4^ Clinical Medical College Shenzhen University Shenzhen China

**Keywords:** anterior cruciate ligament reconstruction, anterior cruciate ligament tear, knee injuries, ligament augmentation and reconstruction system, osteoblasts, vancomycin

## Abstract

**Purpose:**

In this study, we aimed to characterize the elution profile of the ligament augmentation and reconstruction system (LARS) immersed in different concentrations of vancomycin using different immersion methods and determine whether the amount of vancomycin released was lower than the toxic concentrations for osteoblasts and chondroblasts.

**Methods:**

The LARS was presoaked with 5, 2.5 or 1.25 mg/mL vancomycin solutions or wrapped in presoaked sterile gauze. After 10 min, the rinsed and unrinsed LARS samples were eluted in 100 mL agitated 37°C phosphate‐buffered saline. An ultraviolet spectrophotometer was used to analyze 1 mL samples taken after 10 min and 0.5, 1, 6, 12, 24h.

**Results:**

Initially, no hysteresis was observed with vancomycin release into the solution at the tested conditions. The LARS elution profiles for different concentrations of the vancomycin solutions varied significantly. The amount of vancomycin released after 24 h was 9.10 ± 1.21, 5.29 ± 0.63 and 2.28 ± 0.59 mg for the 5, 2.5 and 1.25 mg/mL solutions, respectively. The amount of vancomycin released in the soaked group was significantly higher than in the rinsed and wrapped groups. The released amounts of vancomycin were below the toxic concentrations for osteoblasts and chondrocytes.

**Conclusion:**

Soaked LARS can act as a reservoir for vancomycin, with the amount released and the elution profile dependent on rinsing, soaking solution concentration and soaking method. The eluted concentrations of vancomycin were lower than those previously reported for osteoblast and chondrocyte toxicity and higher than the minimal inhibitory concentrations for *Staphylococci*.

**Level of Evidence:**

N/A.

## Graphical abstract

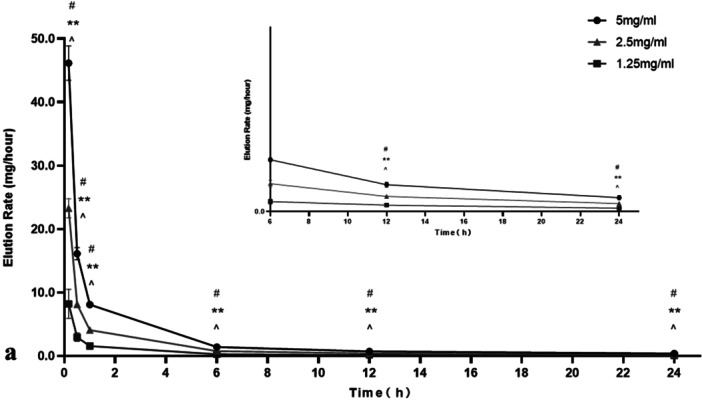

**Abbreviations**
ACL: anterior cruciate ligament
ACLR: anterior cruciate ligament reconstruction
LARS: ligament advanced reinforcement system
PBS: phosphate‐buffered saline

## INTRODUCTION

Anterior cruciate ligament (ACL) injury represents a prevalent medical condition across various age groups of patients, most of which are attributed to noncontact mechanisms involving pivoting, cutting or jumping [9, 11, 15, 18, 20, 23]. ACL tears can result in knee instability, decreased sports ability and secondary knee osteoarthritis [1]. In clinical practice, ACL reconstruction (ACLR) is frequently performed arthroscopically, a safe and effective method for restoring knee function and stability [3, 6, 21, 22]. The most used grafts include autografts (hamstring tendon, bone‐patellar tendon‐bone and quadriceps tendon), allografts and artificial ligaments (ligament augmentation and reconstruction system [LARS]), each with its advantages and disadvantages [7, 14, 15]. The LARS has similar clinical efficacy as autografts and allografts, with a faster recovery rate and no donor site complications, and has been widely used in many countries [5, 19, 20]. Post‐ACLR infection is a rare but severe complication that can result in cartilage degeneration in the knee joint and significantly affect postoperative clinical outcomes [16]. A previous study demonstrated that the rate of post‐ACLR infection is 0.77% [2]. Vancomycin is an effective antibiotic against gram‐positive cocci that inhibits the synthesis of bacterial cell walls and has been widely used in orthopaedic surgery. Vertullo et al. [8] proposed the use of vancomycin for the pre‐immersion of autologous tendon grafts. This method has significantly reduced the rate of postoperative infections by eluting at concentrations significantly higher than the lowest inhibitory concentration of *Staphylococci* while preventing osteoblast and chondrocyte toxicity. With the increasing implementation of LARS in ACLR, there is growing concern among surgeons regarding the potential for severe complications arising from postoperative infections. Vancomycin, a well‐established antibiotic, is frequently employed as a prophylactic agent to prevent such infections. Given the critical need to optimize infection prevention strategies, it is essential to investigate the application of vancomycin in the context of LARS. Specifically, research is required to elucidate its elution properties, optimal elution methods, and antimicrobial efficacy. Such findings would provide evidence‐based guidance to clinicians for intraoperative use, potentially leading to a significant reduction in postoperative infection rates. Second, the capacity of LARS to effectively replicate the release properties of vancomycin in tendons remains unexplored. This study aims to address this critical research gap.

Therefore, in this study, we aimed to characterize the elution profiles of LARS using different vancomycin concentrations and immersion methods and determine whether the released amount of vancomycin is above the effective inhibitory concentration for *Staphylococci* and below the toxic concentration for osteoblasts and chondrocytes.

## MATERIALS AND METHODS

### Materials

The materials used in this study included: LARS (MOVMEDIX), vancomycin (HISUN), phosphate‐buffered saline (PBS, VivaCell) and GENESYS 50 (Thermo Fisher).

### Preparation of vancomycin soaking solutions

Vancomycin solutions were prepared by dissolving 500 mg of vancomycin hydrochloride in PBS and subsequently diluting to achieve final concentrations of 5, 2.5 and 1.25 mg/mL.

### Soaking of the LARS

LARS samples were soaked in vancomycin solutions at concentrations of 5, 2.5 and 1.25 mg/mL for 10 min and, thereafter, were either rinsed with 5 mL of PBS before the elution study or left unrinsed. Sterile gauze swabs (10 × 10 cm^2^*eight ply) were soaked in vancomycin solution for 1 min. The LARS samples were tightly wrapped in soaked gauze swabs and left to stand for 10 min.

### Vancomycin elution from LARS

The elution protocol was as previously described with some modifications [8]. The presoaked LARS samples were eluted in covered chambers containing either 50 or 100 mL of PBS (pH 7.4) at a constant temperature of 37°C and agitated continuously. At 10 min and 0.5, 1, 6, 12, 24h, 1 mL of the solution was taken and stored immediately at −20°C. To maintain the original volume, an equivalent volume of PBS was added to each chamber. The samples were analyzed using GENESYS 50 within 7 days of collection. Vancomycin was then detected at 280 nm.

### Data processing

The release of vancomycin from each LARS during specified sampling intervals was computed. For each sampling interval, the vancomycin concentration of every sample was measured and multiplied by the fixed chamber volume (either 50 or 100 mL). This multiplication yielded the total quantity of vancomycin released into the chamber over that particular interval. The cumulative amount of vancomycin released by each LARS was determined following established methodology [8].

### Statistical analysis

Quantitative data are presented as mean ± standard deviation (SD). Statistical differences were calculated using the Kruskal–Wallis test and Mann–Whitney *U* test using GraphPad Prism 9.5.0 software and SPSS 26.0 when appropriate. *p* Values < 0.05 were considered statistically significant and illustrated by *p < 0.05, **p < 0.01, ***p < 0.001, ^#^ *p* < 0.05, ^##^ *p* < 0.01, ^###^ *p* < 0.001, ^*p* < 0.05, ^^P < 0.01 and ^^^*p* < 0.001, respectively.

## RESULTS

**Figure 1:**
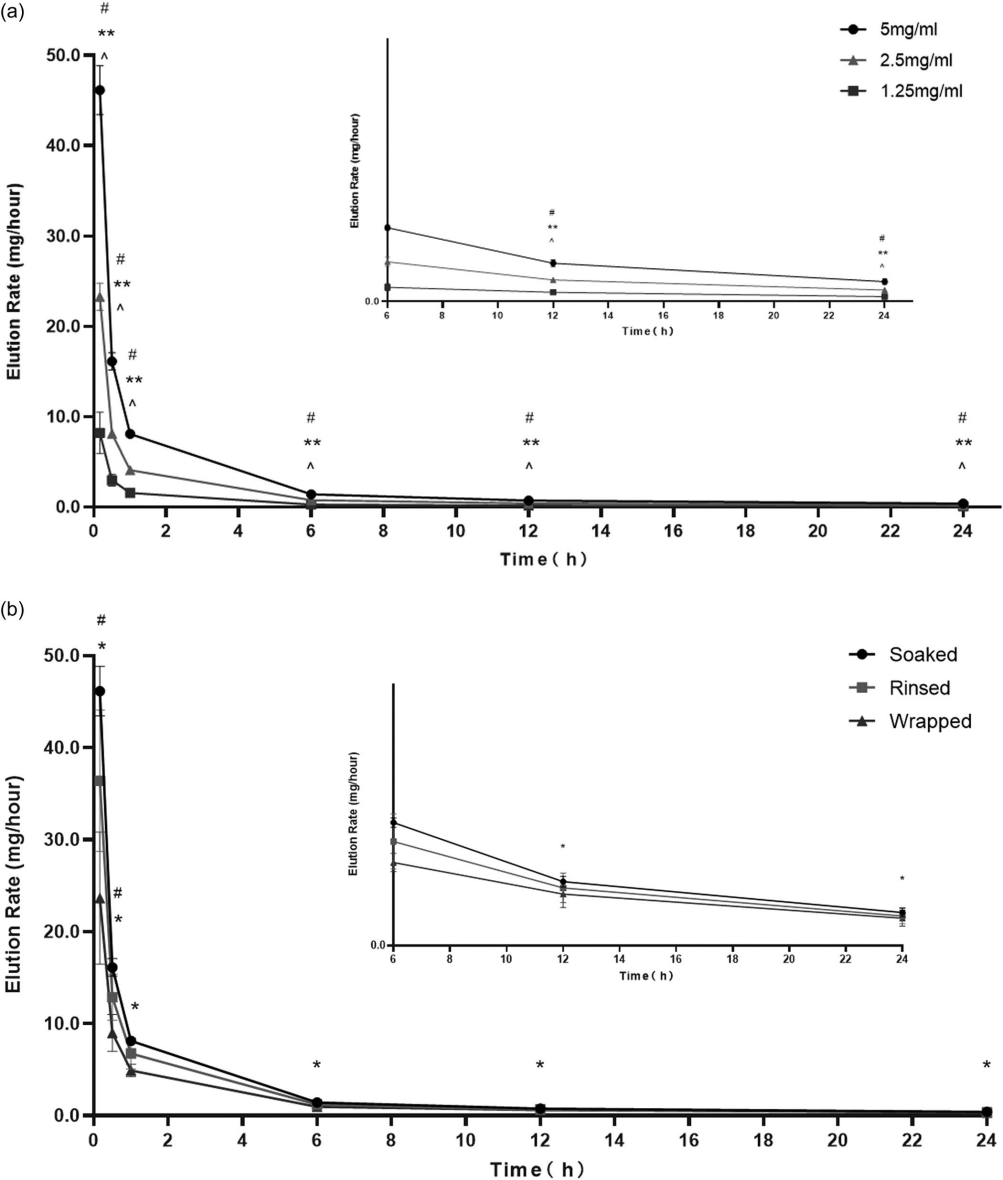
(a and b) No hysteresis was observed when the vancomycin was released into the solution at the start of the test. The maximum elution rates were observed at 10, 30 min and 1 h sampling intervals under all study conditions.

No hysteresis was observed when the vancomycin was released into the solution at the start of the test, which means that LARS can actively and rapidly release vancomycin. The maximum elution rates were observed at 10, 30 min and 1 h sampling intervals under all study conditions (Figure 1a,b).

The cumulative release of vancomycin demonstrated an upward trend across all sampling intervals as the concentration of the vancomycin solution increased. Specifically, after 24 h, the quantities of vancomycin released were 9.10 ± 1.21, 5.29 ± 0.63 and 2.28 ± 0.59 mg for solutions with concentrations of 5, 2.5 and 1.25 mg/mL, respectively. These findings indicate a dose‐dependent relationship between solution concentration and vancomycin release.

**Figure 2:**
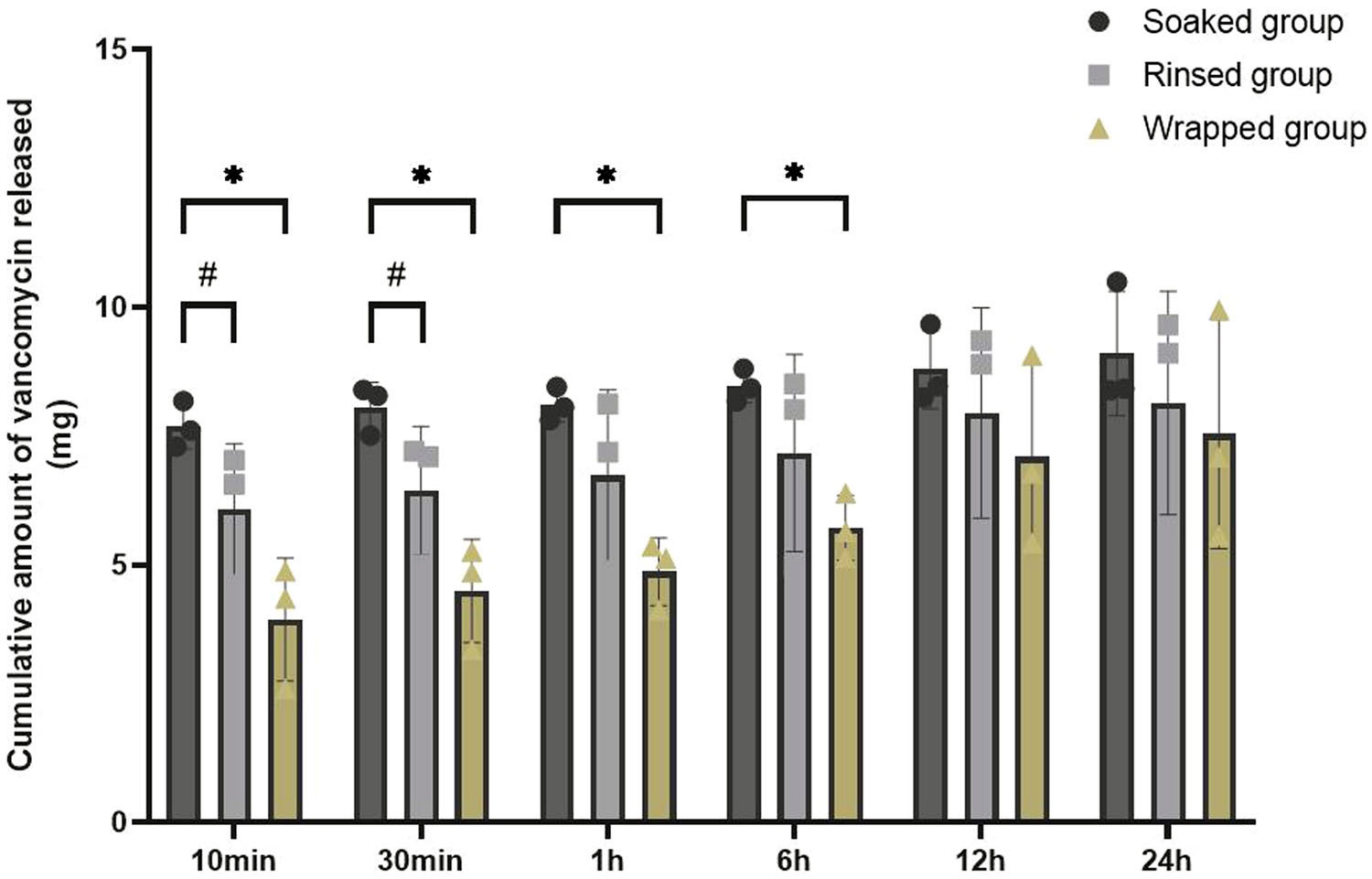
After 30 min, no statistically significant difference was observed in the rate of vancomycin elution between the soaked and rinsed groups. After 6 h, the elution rates of the soaked and rinsed groups became equivalent to those of the wrapped group.

These results demonstrated that the amount of vancomycin released from the soaked group was significantly higher than that released from the rinsed and wrapped groups. The amount of vancomycin in the soaked group was higher just in the first 10 min, compared to 30 min, 6 and 24 h (Table 1). After 30 min, no statistically significant difference was observed in the rate of vancomycin elution between the soaked and rinsed groups. After 6 h, the elution rates of the soaked and rinsed groups became equivalent to those of the wrapped group (Figure 2).

**Table 1:** The amount of vancomycin released from the soaked group was significantly higher than that released from the rinsed and wrapped groups.

Vancomycin concentration (mg/mL)	Group	Cumulative amount of vancomycin released (mg)^a^					
		10 min	30 min	1 h	6 h	12 h	24 h
1.25	Soaked	1.37 ± 0.38	1.49 ± 0.33	1.57 ± 0.40	1.64 ± 0.39	2.13 ± 0.52	2.28 ± 0.59
2.5	Soaked	3.88 ± 0.25	4.06 ± 0.17	4.08 ± 0.19	4.56 ± 0.54	4.96 ± 0.53	5.29 ± 0.63
5	Soaked	7.69 ± 0.45	8.06 ± 0.48	8.10 ± 0.33	8.47 ± 0.32	8.79 ± 0.77	9.10 ± 1.21
5	Rinsed	6.07 ± 1.28	6.43 ± 1.25	6.73 ± 1.67	7.17 ± 1.91	7.94 ± 2.04	8.14 ± 2.17
5	Wrapped	3.94 ± 1.19	4.49 ± 1.00	4.86 ± 0.66	5.72 ± 0.63	7.09 ± 1.84	7.53 ± 2.22

Abbreviation: SD, standard deviation.

a Values are expressed as mean ± SD.

After 24 h, the cumulative concentrations of vancomycin were 91.0 ± 12.1, 16 ± 1 and 9 ± 3 µg/mL for the 5, 2.5 and 1.25 mg/mL vancomycin soaking solutions, respectively. All the released amounts of vancomycin were below the concentrations toxic to osteoblasts (>1000 µg/mL) and chondrocytes (>6.125 mg/mL) [14, 18].

## DISCUSSION

Post‐ACLR infection is one of the most devastating knee complications, which may not only lead to high readmission rates and poor knee function but may also have a significant negative effect on the patient's psychological health and medical expenses, especially in athletes, as it might affect their sports careers [2]. However, prophylactic graft saturation with vancomycin has reportedly reduced infection rates. The release of drugs and elution profiles for vancomycin from tendons depend on several factors including rinsing procedures, tendon dimensions, and the concentration of the vancomycin solution employed for soaking [8]. However, no studies have been published regarding the LARS immersed in vancomycin solution. Further, it is unknown whether the reported method can be used to reduce infection rates. Our results demonstrated that LARS can serve as a reservoir for vancomycin release with optimal biocompatibility, enabling the efficient release of vancomycin inside the joint. It can achieve effective inhibitory concentrations against bacteria without causing toxicity to chondrocytes. Therefore, the findings of our study offer valuable guidance for clinical decision‐making aimed at reducing the incidence of postoperative infections following ACLR.

The results of this study showed that LARS was effective in releasing vancomycin after it had been soaked in different concentrations of vancomycin solution. The release rate of vancomycin peaked within 10 min of elution, after which it gradually declined to a residual amount that was released after 24 h. This suggests that LARS prepared using the described protocol is a reservoir for vancomycin release. The elution of vancomycin is not sustained for long periods of time and therefore should not pose a risk to osteoclasts and chondrocytes owing to accumulation of the drug locally. This release profile is consistent with the findings of Vertullo et al. [8], indicating that LARS has an almost identical structure and drug absorption and release properties as natural tendons.

Different handling methods have been demonstrated to be effective in reducing the amount of vancomycin released into the joint cavity, with the greatest efficacy observed after 10 min. Rinsing removes excess vancomycin from the soaking solution that has accumulated on the surface of the LARS. Following rinsing, vancomycin elution from the LARS was observed. Similarly, the use of gauze to wrap the LARS resulted in a further reduction in the amount of vancomycin released. The detection of increasing amounts of vancomycin in the solution throughout the experiment, suggests potential distribution of vancomycin within LARS. This finding is further supported by the nonlinear elution rates noted across varying concentrations of the soaking solutions. Different concentrations of vancomycin and soaking methods have proven effective in the release of vancomycin, thereby achieving safe and efficacious bactericidal concentrations. This finding serves as a valuable reference for clinical decision‐making, ensuring the minimal use of vancomycin while effectively preventing infections. Nonetheless, it is crucial to acknowledge that this study utilizes an in vitro model designed to simulate the joint cavity; thus, further investigations involving animal and human subjects are essential to accurately guide clinical applications and evaluate the associated benefits and risks of vancomycin use in this context.

However, elution rates declined rapidly after 1 hour, indicating that vancomycin is unlikely to accumulate significantly over prolonged periods due to ongoing elution, even in a static environment. This reduction in elution rate can be attributed to diminishing vancomycin availability within the LARS, thereby affecting the concentration gradient and favouring diffusion into the chamber rather than the saturation of the solution within the chamber. Notably, all measured vancomycin concentrations across tested conditions remained well below its documented solubility threshold, underscoring its high water solubility [12].

Furthermore, the vancomycin in LARS is largely released within a 24‐h interval, and the amount of vancomycin released is below the concentration that is toxic to osteoblasts (>1000 µg/mL) and chondrocytes (>6.125 mg/mL) [13, 17]. Consequently, vancomycin reduces the incidence of post‐ACLR infection without having toxic effects on the tissues within the joint cavity, thereby influencing the healing process. However, this hypothesis requires further validation using in vivo study.

LARS is a new‐generation synthetic artificial ligament with advantages, such as brief hospitalization, less invasiveness, reduced period of immobilization, absence of muscle atrophy, and rapid functional recovery (approximately 1–2 months), with mild postoperative pain and swelling in 90% of patients [4, 10]. According to our study, LARS has almost identical structures, and its drug absorption and release properties are similar to those of natural tendons, providing theoretical support for its widespread application. There is a paucity of guidelines in the literature regarding the use of vancomycin for the prevention of post‐ACLR infection. Furthermore, the concentration of vancomycin used by surgeons varies considerably. This study provides a clinical reference for the use of vancomycin in LARS‐based ACLR. All three methods used were effective and safe for killing bacteria, and surgeons could choose one of them according to their preferences.

This study has some limitations. This study presents an in vitro model simulating the release of vancomycin from LARS into a chamber, aiming to mimic in vivo conditions. However, the human body possesses intricate mechanisms for regulating temperature and pH levels. Notably, the knee joint cavity following ACLR exhibits challenges in maintaining a constant temperature and pH compared to in vitro models. Numerous factors contribute to the degree of mobility experienced in the knee post‐surgery. An in vitro model cannot fully simulate the microenvironment of the joint cavity. Therefore, further studies in animals and humans are required.

## CONCLUSION

Soaked LARS can act as a reservoir for vancomycin, with the amount released and the elution profile dependent on rinsing, soak solution concentration, and soaking method. The elution concentrations of vancomycin were lower than those previously reported for osteoblast and chondrocyte toxicity and higher than the minimal inhibitory concentrations for *Staphylococci*. This study confirms that LARS exhibits structural characteristics and drug absorption and release properties comparable to those of natural tendons. Furthermore, the different concentrations of vancomycin and the soaking methods are both effective and safe for bacterial eradication. This finding addresses a significant gap in the existing literature and holds the potential to guide clinical decision‐making, thereby contributing to a reduction in the incidence of postoperative infections among patients. However, it is essential that further studies be conducted in animal and human models to validate these results and explore their clinical implications.

## AUTHOR CONTRIBUTIONS

Yong Luo contributed to the conception and design of the study, data collection, and drafting of the manuscript. MingYang Zou contributed to the experimental design and manipulation, data collection and preliminary analysis. XinTao Zhang critically reviewed and revised the manuscript for important intellectual content. SuFen Ye, XianCheng Huang and JiaTong Li carried out validation and replication experiments to ensure the reproducibility and stability of the experimental results. Tian You provided overall project leadership and direction, coordinated the efforts of the authors, and also provided critical resources and equipment support necessary for the experiments. All authors reviewed and approved for publication.

## CONFLICT OF INTEREST STATEMENT

The authors declare no conflict of interest.

## ETHICS STATEMENT

This article does not contain any studies with human participants or animals performed by any of the authors. Consent for publication: Not applicable.

## Data Availability

The datasets used and analyzed during the current study are available from the corresponding author on reasonable request.
